# BMP4, a strong better prognosis predictor, has a subtype preference and cell development association in gliomas

**DOI:** 10.1186/1479-5876-11-100

**Published:** 2013-04-16

**Authors:** Zhaoshi Bao, Chuanbao Zhang, Wei Yan, Yanwei Liu, Mingyang Li, Wei Zhang, Tao Jiang

**Affiliations:** 1 Department of Neurosurgery, Beijing Tiantan Hospital, Capital Medical University, Beijing, 100050, China; 2 Beijing Neurosurgical Institute, Beijing, 100050, China

**Keywords:** BMP4, Cell development, Survival, Glioma

## Abstract

**Background:**

The bone morphogenetic family proteins (BMP) are phytogenetically conserved proteins, which are essential for embryonic development. The key regulatory subunit, the bone morphogenetic protein 4 (BMP4), is overexpressed and associated with tumor metastasis in a variety of cancers. However, the prognostic and molecular features of gliomas with BMP4 expression is still unclear.

**Methods:**

We obtained whole genome mRNA expression microarray data of 220 glioma samples of all grades from Chinese Glioma Genome Atlas (CGGA) database (http://www.cgga.org.cn) as discovery set. Of the 123 high-grade gliomas in this set, 33 Grade III tumors and 88 GBMs were analyzed by Kaplan-Meier method. Immunohistochemistry was used for validating the expression of BMP4 in another 77 glioma samples. Three additional datasets were obtained as validation sets. Gene ontology (GO) analysis and gene set variation analysis (GSVA) were used for functional annotation of BMP4.

**Results:**

In the discovery set, BMP4 overexpression was significantly associated with low grade as well as the lower mortality of high-grade gliomas in survival analysis (log-rank, p<0.05 in GBM patients and p<0.01 in anaplastic gliomas, respectively). BMP4 also showed a Proneural subtype, G1 subtype and Isocitrate Dehydrogenase 1 (IDH1) mutation preference and cell development association. The results of validation 4 datasets showed similar findings. The overexpression of BMP4 was also detected in low grade gliomas compared to the high grade ones by immunohistochemistry (p<0.05, chi-square test).

**Conclusion:**

BMP4 expression was independently associated with grade and good prognosis in grade III and grade IV gliomas, suggesting BMP4 as a novel biomarker with potential important therapeutic implications.

## Background

Glioma is the most common type of brain tumor and is an important cause of cancer related mortality among adults and children [1]. It can be divided into low grade glioma (LGG) and high grade glioma (HGG) depending on the malignancy. The median survival of patients with primary glioblastoma (GBM), the most malignant and frequent type of glioma, is approximately 1 year. But because of the heterogeneity of cancer, it varies remarkably from <1 week to >3 years after diagnosis [2], suggesting the limitations of the current diagnostic, predictive and prognostic markers and better therapeutic strategies are in the urgent need.

The introduction of molecular biomarkers in the management of patients with cancer may improve their clinical outcomes. The bone morphogenetic family proteins (BMP) are phytogenetically conserved proteins, which are essential for embryonic development [3,4]. Their key regulatory subunit, the bone morphogenetic protein 4 (BMP4), is overexpressed and associated with pathogenesis and metastasis in a variety of cancers [5-7]. However, the prognostic and molecular features of gliomas with BMP4 expression is still unclear.

In this study, We obtained whole genome mRNA expression microarray data of 220 glioma samples of all grades from Chinese Glioma Genome Atlas (CGGA) database (http://www.cgga.org.cn) as discovery set [8] and 3 additional previously published datasets as validation sets. After studying the expression level of BMP4 in these samples, we analyzed the prognostic value of it. The expression difference was validated in another 77 glioma samples from Chinese Glioma Tissue Database (CGTD) by Immunohistochemistry. We also performed function annotation of BMP4 by GO analysis and GSVA, which revealed its correlation with cell development, differentiation and biogenesis.

## Methods

### Datasets used in this study

Whole genome mRNA expression microarray data and clinical information of 220 glioma samples of all grades from Chinese Glioma Genome Atlas (CGGA) database [8] (http://www.cgga.org.cn) were obtained as discovery set and 202 glioma expression files from the cancer genome atlas (TCGA) database [9] (http://cancergenome.nih.gov), the Repository for Molecular Brain Neoplasia Data (REMBRANDT, https://caintegrator.nci.nih.gov/rembrandt/) and GSE16011 data [10] (http://www.ncbi.nlm.nih.gov/geo/query/acc.cgi?acc=GSE16011) were obtained as validation sets.

### Gene ontology (GO) analysis of BMP4 associated genes

After Pearson correlation analysis, gene ontology analysis of the positively correlated genes (r>0.4, p<0.05) were analyzed by DAVID (http://david.abcc.ncifcrf.gov/home.jsp).

### GSVA with BMP4 expression

Gene set variation analysis with BMP4 expression was analyzed by GSVA package [11] of R [12]. Gene list was obtained from GSVAdata package [13].

### Immunohistochemistry (IHC)

Briefly, Immunoperoxidase staining for BMP4 (Abcam, ab39973) were performed following the standard protocol recommended by the manufacturer. Each slide stained for and BMP4 was individually reviewed and scored by two independent observers. Discrepancies in scoring between the two observers were resolved by additional review of the specimens and discussion between the reviewers until a consensus was achieved. Approximately 15-20 fields at 400× magnification were analyzed per specimen. The proportion of positively stained tumor cells was graded as follows: 0, no positive tumor cells; 1, <5% positive tumor cells; 2, 5-20% positive tumor cells; and 3, >20% positive tumor cells. The intensity of staining was recorded on a scale of 0 (no staining), 1 (weak staining, light yellow), 2 (moderate staining, yellowish brown) and 3 (strong staining, brown). The staining index was calculated as follows: staining index = staining intensity × tumor cell staining grade. High BMP4 expression was defined as a staining index score ≥4, while low expression was defined as a staining index <4.

### Statistical analysis

For molecular subtype annotation of the 4 datasets, we applied prediction analysis of microarrays (PAM) as previously reported [8]. Quantitative results were shown as mean ± standard deviation. The difference of BMP expression was tested by the Student t-test in microarray data and by chi-square test in IHC results. Overall survival time (OS) was calculated from the date of diagnosis until death or the last follow-up. The survival curve of patients with high or low expressed BMP4 was calculated with the Kaplan-Meier method and the difference was analyzed using the two-sided log-rank test. A p-value < 0.05 was considered statistically significant. All the data analysis was performed in GraphPad Prism and R.

Written informed consent was obtained from the patient for publication of this report and any accompanying images. The study was performed with the approval of Ethics Committee of Capital Medical University and was in compliance with the Helsinki Declaration.

## Results

### Characteristics of patients included

Of the 220 glioma patients in the training set, there were 97 grade II gliomas (astrocytomas, oligodendrogliomas, and oligoastrocytomas), 34 grade III gliomas (anaplastic astrocytomas, anaplastic oligodendrogliomas, and anaplastic oligoastrocytomas) and 89 grade IV gliomas (GBMs). Clinical information (age, gender, preoperational Karnofsky Performance Scale (KPS) score, and treatment) were obtained from medical records of CGGA database, which were listed in Table 1.

**Table 1:** Clinical characteristics of 220 glioma patients

	**Grade II**	**Grade III**	**Grade IV**
Median age	38	39	46
Male %	55.7	52.9	58.4
Median KPS	90	80	80
Median OS (days)	ND	633	420
ND, not determined			

### BMP4 was overexpressed in low-grade gliomas

**Figure 1:**
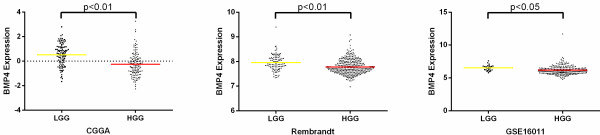
**The expression difference of BMP4 in LGG and HGG of CGGA and other two validation datasets.** A single spot was the expression value of BMP4 of an individual patient. Lines in the middle were the mean expression value.

After screening of differently expressed genes in the discovery dataset, we found that BMP4 was in the list of genes significantly differently expressed between LGGs and HGGs. Both in CGGA and other 3 validation datasets, the expression of BMP4 in LGG was higher than that in HGG (Figure 1).

### The expression level of BMP4 was validated in an independent group of patients by IHC

**Figure 2:**
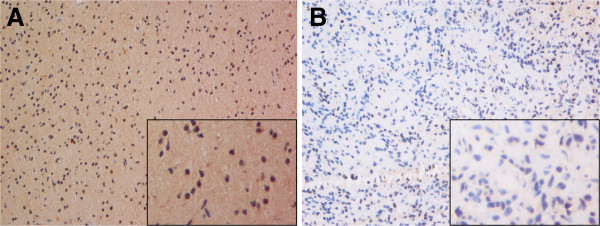
**IHC images of 2 distinct patients. A** (LGG patient), showed high expression of BMP4 in cytoplasm; **B** (HGG patient), showed low expression of BMP4. Magnification: larger images, ×200; smaller images, ×400.

We further validated the protein expression level of BMP4 in an independent group of 77 glioma patients by IHC (Figure 2). Similar to the findings above, BMP4 showed a higher expression status in LGGs than that in HGGs (p<0.05, chi-square test). Thus, BMP4 expression showed a LGG preference both in mRNA and protein level.

### BMP4 was a better prognostic marker in anaplastic gliomas and glioblastomas

**Figure 3:**
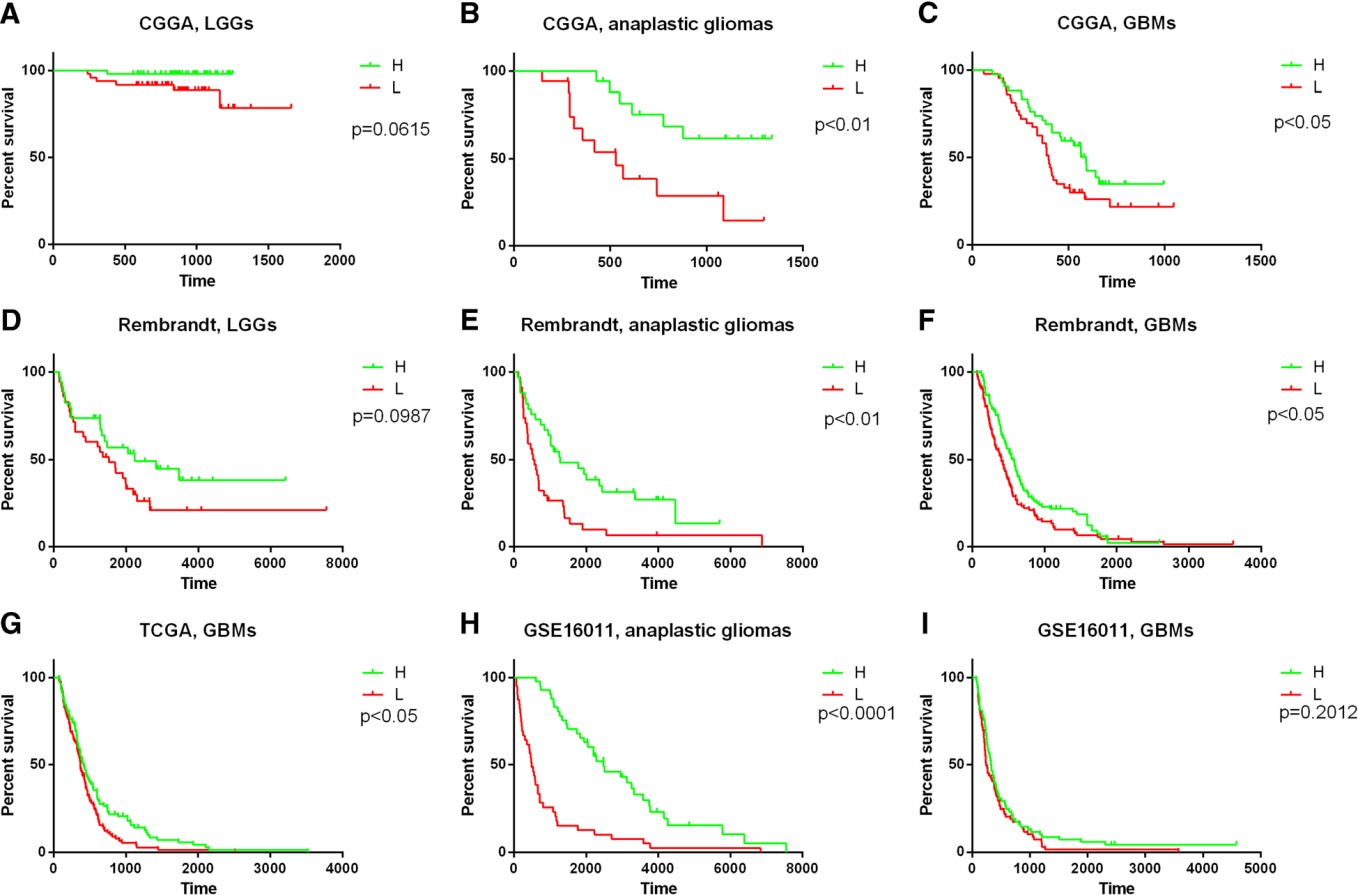
**The prognostic value of BMP4 in CGGA and validation datasets.** Except for GBM patients in GSE16011 dataset (**I**), according to BMP4 expression level, patients with anaplastic or GBM could be divided into two groups with significantly different prognosis, respectively (**B**, **C** anaplastic gliomas and GBMs in CGGA data; **E**, **F**, anaplastic gliomas and GBMs in Rembrandt data; **G**, GBMs in TCGA data; **H**, anaplastic gliomas in GSE16011 data). For LGG patients, there is only marginal p value (**A**, LGGs in CGGA data; **D**, LGGs in Rembrandt data). **H**, higher expression of BMP4 than the median one. L, lower expression of BMP4 than the median one.

We confirmed the prognosis of the 220 patients, and got 216 patients for further prognosis analysis (the prognosis of 2 patients were not available and the OS of another 2 patients were too short which might due to other complications other than glioma). As is shown in Figure 3, both anaplastic glioma (Figure 3B) and GBM (Figure 3C) patients with high or low expression of BMP4 had considerable different prognosis. But there was only a marginal p value in LGGs (Figure 3A). The results were similar in the validation set (Figure 3D-I). Therefore, BMP4 was a better prognostic marker in anaplastic gliomas and glioblastomas. The high or low expression was defined as higher or lower than the median individual in each grade.

### BMP4 expression showed a subtype preference

**Figure 4:**
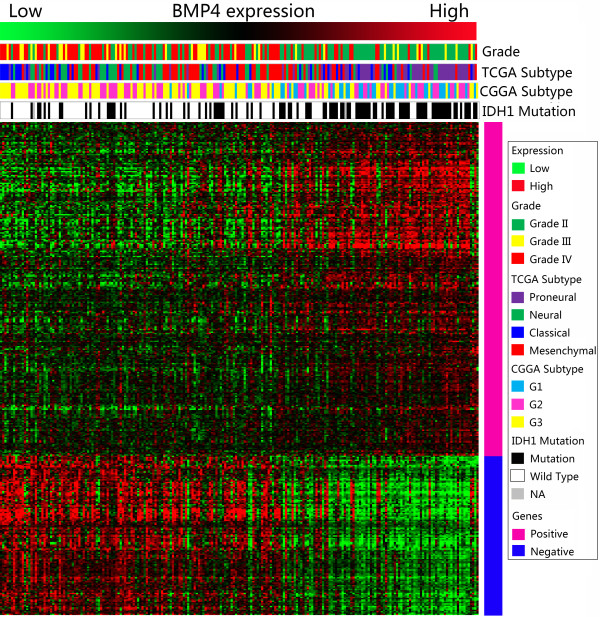
**BMP4 expression showed a Proneural, G1 subtypes and IDH1 mutation preference.** For each patient, TCGA and CGGA subtype were annotated as previously reported and listed in the upper part as well as the IDH1 mutation status, which was obtained from CGGA database. The positively and negatively correlated genes were showed in the lower part (marked pink and blue in the right, respectively). NA, not available.

**Figure 5:**
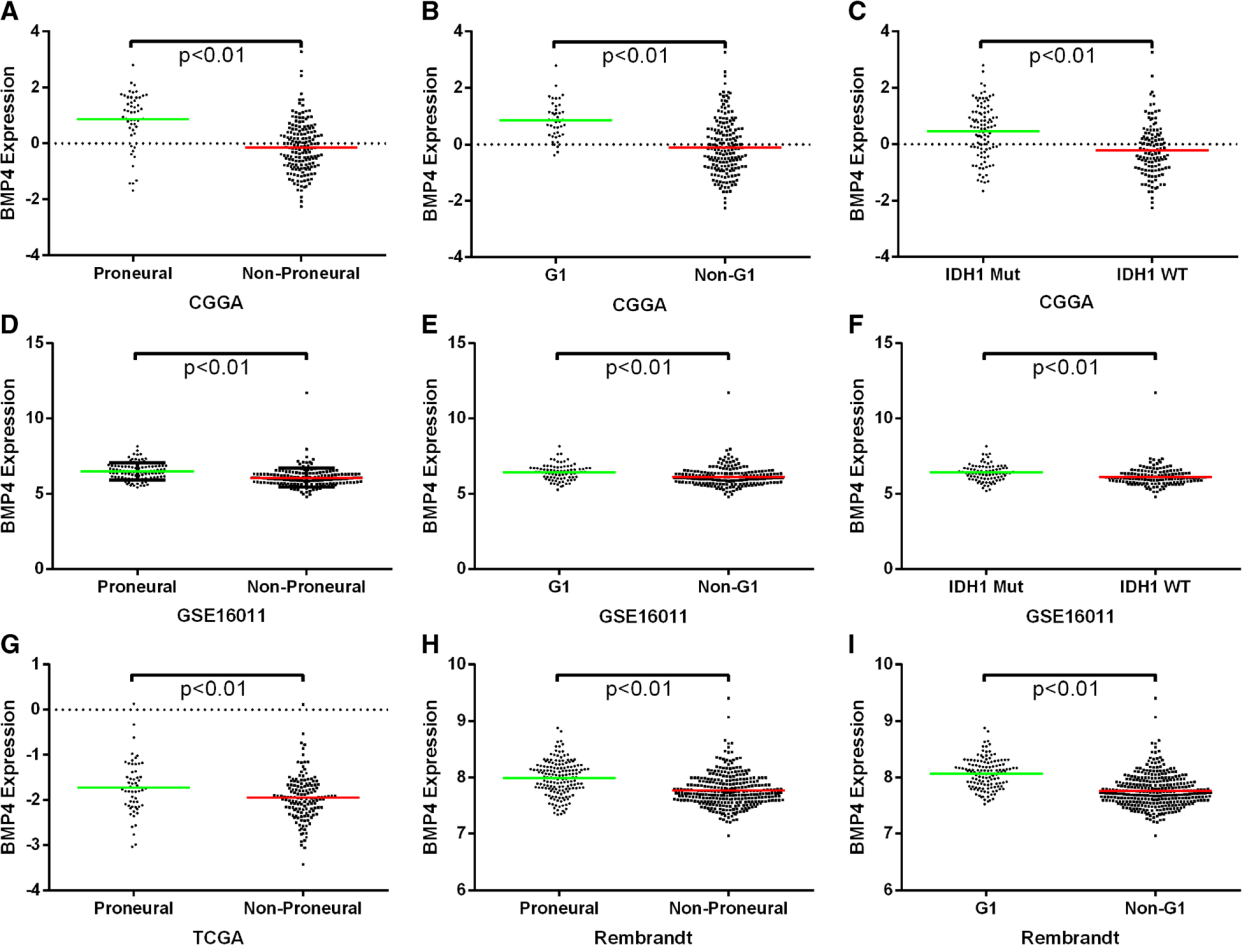
**The BMP4 expression was significantly different for different TCGA and CGGA subtypes and IDH1 mutation status. A**, **D**, **G** and **H** showed BMP4 had a Proneural preference. **B**, **E** and **I** showed BMP4 had a G1 preference. **C** and **F** showed that patients with IDH1 gene mutation also showed higher expression of BMP4 than those with wild-type IDH1 gene. A single spot was the expression value of BMP4 of an individual patient. Lines in the middle were the mean expression value.

As BMP4 showed association with LGGs and better prognosis, we screened its expression in different molecular subtypes of gliomas. As previously reported, we annotated the 4 datasets by TCGA and CGGA classification systems by PAM [8,9]. BMP4 showed a Proneural and G1 subtype preference. Patients with IDH1 gene mutation also showed higher expression of BMP4 than those with wild-type IDH1 gene (Figure 4). The expression difference was shown in Figure 5.

### BMP4 was associated with cell development

After Pearson correlation analysis of the data from CGGA, the significantly positively correlated genes (Pink marked genes in Figure 4) were used for GO analysis. The top 20 GO terms listed in Table 2 showed that BMP4 was associated with cell development, differentiation and biogenesis.

**Table 2:** Top 20 GO terms of BMP4 positively associated genes

**GO term**	**Biological process**
GO:0030509	BMP signaling pathway
GO:0048710	regulation of astrocyte differentiation
GO:0045685	regulation of glial cell differentiation
GO:0014013	regulation of gliogenesis
GO:0060284	regulation of cell development
GO:0048712	negative regulation of astrocyte differentiation
GO:0007386	compartment specification
GO:0007178	transmembrane receptor protein serine/threonine kinase signaling pathway
GO:0006350	transcription
GO:0010721	negative regulation of cell development
GO:0016202	regulation of striated muscle tissue development
GO:0048634	regulation of muscle development
GO:0050767	regulation of neurogenesis
GO:0045449	regulation of transcription
GO:0045686	negative regulation of glial cell differentiation
GO:0014014	negative regulation of gliogenesis
GO:0051960	regulation of nervous system development
GO:0042474	middle ear morphogenesis
GO:0007507	heart development
GO:0007389	pattern specification process

### The positive correlation of astrocytic genes and BMP4

**Figure 6:**
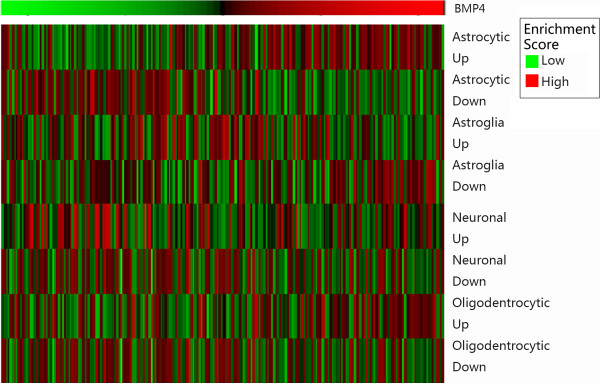
**Gene set variation analysis with BMP4 expression.** Gene expression signatures of oligodendrocytes, astrocytes, neurons, and cultured astroglial cells were generated from GSVAdata package. The BMP4 expression went higher from left to right. A high enrichment score indicates positive correlation with BMP4 expression and a low enrichment score indicates the reverse.

As GO analysis showed that BMP4 had a tight association with cell development, differentiation and biogenesis, especially astrocytes, we performed GSVA with BMP4 expression (Figure 6). Genes up- and down-regulated in astrocytes went positively and negatively with BMP4 expression. Meanwhile, it has been reported that Proneural gliomas are characterized by oligodentrocytic genes which is in concordance with the present study [9]. Oligodentrocytic genes were accumulated in patients with higher BMP4 expression, which was preferentially expressed in Proneural gliomas.

## Discussion

Glioma is the most common lethal intracranial tumor in adults. Even after years of efforts in developing and improving therapeutic strategies, the glioma patients still have a poor survival time. As the identification of novel biomarkers and new molecular classification systems, we really see the dawn of a new era.

The bone morphogenetic family proteins (BMP) are highly conserved proteins, which are essential for embryonic development. Since potent developmental regulators are frequently disrupted in cancer [14], it is to be expected that BMP4 also contributes to tumor development. In recent years, important new advances has been generated on the contribution of BMP family members, such as BMP4, in cancer pathogenesis. Firstly, BMP4 gene variants have been shown to predispose to colorectal cancer [15]. Meantime, the expression level of BMP4 are frequently altered in many tumor types [5-7,16,17]. Both in vivo and in vitro studies have demonstrated the role of BMP4 on suppression of cell growth [18,19], induction to migration, invasion and epithelial-mesenchymal transition [20,21], which are associated with cancer metastasis and progression.

There are only limited reports on the role of BMP4 in gliomas focusing on the anti-proliferation effect of BMP4 to stem-like cells [22-25] and GBM cell lines [26].In the present study, we found that BMP4 was overexpressed in LGGs. The expression difference was validated by microarray data from other datasets and IHC results from an independent group of patients from CGTD. These results showed that BMP4 was a potential marker for grading of gliomas.

Considering its effects on growth suppression, BMP4 has been suggested as a possible therapeutic target in cancer cells. Nevertheless, the other functional characteristics of BMP4, especially the promotion of cell mobility, make such strategies less appealing. Improved knowledge of the downstream mediators of BMP4 effects in cancer cells may allow dissection of the different BMP4 induced phenotypes and thereby generation of specific targeted therapies [27].

For prognosis analysis, we generated survival curve of data from CGGA and 3 other datasets by Kaplan-Meier method and the difference was analyzed using the two-sided log-rank test. Patients with higher expression of BMP4 showed a significantly better prognosis in anaplastic gliomas and GBMs. That exhibited the predomination of beneficial effects over detrimental effects of BMP4 in gliomas. Hence, BMP4 showed its capacity to be a better prognostic marker and a therapeutic target. We also found the preference expression of BMP4 in IDH1 mutation patients, Proneural subtype or G1 subtype, which was in concordance with the better prognosis of BMP4 overexpressed patients.

## Conclusions

BMP4 was preferentially expressed in LGGs, IDH1 mutation patients, Proneural subtype and G1 subtype. And it was associated with the better prognosis in grade III and grade IV gliomas, all of which suggested that BMP4 was a novel biomarker with potential important therapeutic implications.

## Competing interests

The authors declare that they have no competing interests.

## Authors’ contributions

ZB and CZ made an equal contribution in data analysis and manuscript planning and writing. WY participated manuscript writing and approved the final version. YL and ML were responsible for IHC. WZ and TJ revised the manuscript critically. All authors read and approved the final manuscript.
